# In Response

**DOI:** 10.4269/ajtmh.2011.10-0591b

**Published:** 2011-03-04

**Authors:** Xia Wang, Paul R. Hunter

**Affiliations:** School of Medicine, Health Policy, and Practice; University of East Anglia; Norwich NR4 7TJ, United Kingdom; E-mail: paul.hunter@uea.ac.uk

Dear Sir:

In their letter, Subaiya and Cairncross make the important point that distance to water source is a potentially very important but under researched issue. Millions of persons, predominantly women and children, spend many hours each week in carrying water, and the health and other impacts of this effort is still not fully understood but could be great.^1^

Subaiya and Cairncross are also correct in pointing out the problems with the available evidence on the association between distance to water source and diarrheal disease. We were careful in our initial report to point out the weaknesses within the included studies, the heterogeneity in effect size and the problems in doing comparisons between studies with different designs and comparison points. It is for this reason that we recommended care until properly designed studies are undertaken. However, as pointed out by Subaiya and Cairncross, our analyses would suggest a health effect at distances much closer than previously thought reasonable from studies of water use with distance to source. If this is association with low distances is proven, then the mechanism is unclear. One possibility may be the differential use of container types.^2^

In any event, the message from Subaiya and Cairncross and ourselves is consistent. There is some evidence that distance to water source impacts on the risk of diarrhea and although the quality of the evidence is not great the health impact is potentially great and deserves further research.
